# An Increased B-Type Natriuretic Peptide in the Absence of a Cardiac Abnormality Identifies Those Whose Left Ventricular Mass Will Increase Over Time

**DOI:** 10.1016/j.jchf.2014.07.012

**Published:** 2015-01

**Authors:** M. Adnan Nadir, Stephen Gandy, Sheila Ireland, Tom MacDonald, Ellie Dow, Graeme Houston, Chim Lang, Allan Struthers

**Affiliations:** Division of Cardiovascular & Diabetes Medicine, Medical Research Institute, Ninewells Hospital, Dundee, Scotland, United Kingdom

**Keywords:** B-type natriuretic peptide, left ventricular mass, primary prevention, hypertension, ACE, angiotensin-converting enzyme, ARB, angiotensin receptor blocker, BNP, B-type natriuretic peptide, BP, blood pressure, CMR, cardiac magnetic resonance, CV, cardiovascular, eGFR, estimated glomerular filtration rate, hs-TnT, high-sensitivity troponin T, LV, left ventricular, LVEDV, left ventricle end-diastolic volume, LVESV, left ventricular end-systolic volume, LVH, left ventricular hypertrophy, LVM, left ventricular mass, LVMI, left ventricular mass index

## Abstract

**Objectives:**

The purpose of this study was to identify the relationship of B-type natriuretic peptide (BNP) with evolution of left ventricular mass (LVM) in optimally treated primary prevention patients.

**Background:**

Patients who have an elevated BNP no cardiac abnormality on echocardiography are common and at increased risk of adverse events. One hypothesis is that an elevated BNP is an early sensitive indicator of who will develop future structural abnormalities such as left ventricular (LV) hypertrophy.

**Methods:**

We identified optimally treated primary prevention patients with no cardiac abnormality at baseline. In particular, they had no myocardial ischemia, LV hypertrophy, LV dysfunction, or left atrial enlargement. They had a diverse range of plasma BNP levels and underwent cardiac magnetic resonance at baseline and 3 years later on a 3-T scanner.

**Results:**

Fifty patients with a diverse range of BNP were studied (with BNP ≤10 pg/ml in 25 patients and >10 pg/ml in 25 patients). LVM increased (+4.7 ± 3.5 g) in 24 patients and decreased (–4.9 ± 2.8 g) in 26 patients (p < 0.01). Blood pressure by 24-h monitoring was virtually identical between those whose LVM increased (systolic blood pressure 122 ± 14 mm Hg) and those whose LVM decreased (systolic blood pressure 121 ± 11 mm Hg, p = 0.77). Plasma BNP was nearly 3 times higher in those whose LVM increased versus those in whom LVM decreased (21 ± 9.6 pg/ml vs. 7.9 ± 3.9 pg/ml, p < 0.01). The c-statistic for BNP was 0.88.

**Conclusions:**

In optimally treated primary prevention patients, plasma BNP levels are able to distinguish between those whose LVM will increase during the next 3 years versus those whose LVM will decrease during the next 3 years. This may explain why individuals with high BNP are at increased risk even if no cardiac abnormality can be detected initially.

B-type natriuretic peptide (BNP) is in widespread use as a “rule-out” diagnostic test in patients with suspected heart failure. However, it is not accurate enough to be a “rule-in” test because it is known to produce many false positive results. False positives are also seen when BNP is used for a different diagnostic purpose. For example, BNP can also be used to identify primary prevention patients who are already harboring silent but potentially lethal cardiac abnormalities. Although it performs well overall (c-statistic 0.78) in this latter regard, false positives are still common, that is, 43% of those in the highest tertile of BNP have no apparent cardiac abnormality on phenotyping (1).

BNP is known to be a very strong independent predictor of a poor (cardiac) prognosis in every population ever examined 2, 3, 4, 5, 6, 7. This even seems to be the case in individuals with high BNP levels but no apparent cardiac abnormality on phenotyping (false positives). Several studies show that BNP levels predict prognosis over and above a wide range of baseline echocardiographic abnormalities, although, as would be expected, each echocardiographic abnormality explained part of the BNP risk 4, 5, 6, 7.

The unexplained extra BNP risk not accounted for by echocardiographic abnormalities may be related to BNP being able to predict *future* but not yet apparent abnormalities in left ventricular (LV) structure or function 5, 8. The hypothesis that these data raise is that one of the drivers for future LV abnormalities is intracardiac pressure and that an early subtle elevation in intracardiac pressure can be picked up by BNP before LV abnormalities are either present or detectable on imaging. We therefore set out to see whether in individuals with no apparent cardiac abnormality at baseline a high BNP value could identify those who would develop a cardiac abnormality during the next few years. We focused particularly on left ventricular mass (LVM) to see whether BNP predicted how this would change during the next few years in a population of treated primary prevention patients.

## Methods

### Study population

This is a substudy of a previously reported larger study from our center (1). The original study recruited 300 primary prevention patients with well-treated primary risk factors between April 2008 and July 2010 from local general practitioner surgeries and from the cardiovascular (CV) risk clinic at Ninewells Hospital, Dundee, United Kingdom. Patients included in the original study were 50 years or older, and were eligible for primary prevention only with no previous known CV events. They had to be stable on therapy for at least 1 year and to have reached target for their primary risk factor, for example, office blood pressure (BP) ≤140/90 mm Hg. We excluded those with previously known CV disease, known renal impairment (estimated glomerular filtration rate [eGFR] <60 ml/min), atrial fibrillation, and significant (defined as more than mild) valvular heart disease. All study subjects underwent clinical assessment, biochemical measurements (including BNP), electrocardiography, transthoracic echocardiography, dobutamine stress echocardiography to detect myocardial ischemia, and 24-hour ambulatory BP measurement.

From the original cohort of 300 primary prevention patients, 62 patients underwent baseline cardiac magnetic resonance (CMR) and 50 of them completed a second CMR after a mean follow-up of 3 years. These 62 patients were selected for 2 reasons: 1) freedom from any silent cardiac target organ damage in the form of either left ventricular hypertrophy (LVH), LV diastolic or systolic impairment, left atrial enlargement, and inducible myocardial ischemia; and 2) a wide range of baseline BNP levels. The reasons for 12 patients having no second CMR scan are shown in Online Figure 1.

### Biochemical assays

Biochemical measurements including BNP and high-sensitivity troponin T (hs-TnT) were made by trained laboratory staff blinded to the clinical and echocardiographic data. BNP was measured using Triage BNP assay (Biosite Inc., San Diego, California). The interassay percentage coefficient of variation was 8.8% to 11.6%. The detection limit was 5 pg/ml and upper measuring limit was 5,000 pg/ml. hs-TnT was measured using a highly sensitive assay on an automated platform (Elecsys E170, Roche Diagnostics, Indianapolis, Indiana) with lower limit of blank (3 ng/l) and interassay percentage coefficient of variation ≤10%.

### Cardiac magnetic resonance

Cardiac magnetic resonance was performed at baseline and at 36 months on a 3-T Magnetom Trio scanner (Siemens, Erlangen, Germany) using body array and spine matrix radiofrequency coils as described in detail previously 9, 10. CMR images were analyzed offline by an independent, blinded, magnetic resonance physicist (S.J.G.) using commercial software (Argus, Siemens Multi-modality Work Platform, version VB 15, Siemens). Electronic region-of-interest contours were placed around endocardial and epicardial LV borders on all CMR image slices at end-diastole and end-systole that were identified to contain 50% or more full-thickness myocardium. Papillary muscles were included in the LVM if the muscle structure was indistinguishable from the myocardial wall, but otherwise assigned to the LV blood pool. The process of contour placement was repeated such that every patient dataset at both time points was analyzed twice to optimize the measurement precision. The intraobserver variability was 2.02% at baseline and 1.97% at follow-up.

### Statistical methods

Data for continuous variables are presented as mean ± SD for normally distributed data and median and interquartile range for nonnormally distributed data. Categorical data are expressed as numbers (%). Comparisons between continuous variables were analyzed using the Student *t* test or Mann-Whitney *U* test, whereas categorical variables were analyzed using chi-square test or Fisher exact test. The primary outcome measure was change in left ventricular mass (ΔLVM) from baseline to follow-up at 3 years. The study population was divided into 2 groups depending on the rise or fall in LVM at follow-up compared with the LVM at baseline. We also divided the study cohort into tertiles based on BNP levels according to a prespecified protocol in 2 ways. The first was by dividing the 50 patients into the cohort’s own BNP tertiles. The second was to use the tertile BNP levels of the original 300 patients: this latter approach was used to avoid bias in the way the patients in this substudy were selected from the full cohort (n = 300). The significance level for the trend across the tertiles was calculated by Jonckheere-Terpstra test and chi-square test. Multivariable models were used to identify the predictors of ΔLVM and to calculate c-statistics, and area under the curve was compared by the DeLong method. All statistical analyses were performed using SPSS for Windows version 20.0 (IBM Corp, Armonk, New York), and a 2-sided probability value of <0.05 was considered to be significant. The Tayside Research and Ethics Committee approved the research protocol, and all study participants provided written informed consent.

## Results

### Baseline characteristics of study population

The baseline characteristics of all 50 patients (mean age 64 years, 64% male) are shown in Online Tables 1 and 2. The primary risk factor was hypertension in 43 of 50 (86%) of patients, whereas 31 of 50 (62%) had a history of dyslipidemia. On average all patients had received treatment for their primary risk factor for greater than 3 years at the time of recruitment and were clinically stable with no overt cardiac symptoms. The majority of the patients were on antihypertensive therapy and greater than two-thirds (39 of 50) received either an angiotensin-converting enzyme (ACE) inhibitor or an angiotensin receptor blocker (ARB). Baseline BP assessment by ambulatory BP recording revealed good BP control (mean BP 119/72 mm Hg), whereas 56% (28 of 50) of the study patients also received a statin. Online Table 2 also shows that the 50 patients without target organ damage who received a CMR were virtually identical to the 148 patients in our index study who also had no target organ damage at baseline and did not undergo CMR at follow-up (1).

### CMR data

Fifty patients completed the follow-up CMR scan, and mean follow-up was 36.3 ± 0.9 months. The average LVM at baseline was 105 ± 24 g and 55 ± 9 g/m^2^ when indexed to body surface area. At follow-up, LVM measured lower than baseline (mean Δ –4.9 ± 2.8 g) in 52% (26 of 50), whereas an increase (mean Δ 4.7 ± 3.5 g) in LVM was seen in 48% (24 of 50) patients. Clinical characteristics of patients with a reduction and an increase in LVM are shown in Table 1, and the change in LV data on CMR are summarized in Table 2. Not surprisingly, LV filling (LV end-diastolic volume) was reduced in those whose LVM increased with time.

**Table 1:** Characteristics of Study Population Stratified by Change in LVM (n = 50)

Variable	LVM Decreased (n = 26)	LVM Increased (n = 24)	Significance∗
Age (yrs)	63.8 ± 6.1	61.5 ± 4.1	0.39
Male/female	18/8 (69%/31%)	14/10 (58%/42%)	0.39
Hypertensive	24 (92%)	19 (80%)	0.40
Dyslipidemia	19 (73%)	12 (50%)	0.08
Duration of treatment (yrs)	3.5 ± 2.4	4.3 ± 4.7	0.55
Smoker	3 (12%)	10 (41%)	0.02
Family history of cardiovascular disease	15 (58%)	16 (67%)	0.55
BMI (kg/m^2^)	26.6 ± 4.0	27.5 ± 3.0	0.19
SBP (mm Hg)	121 ± 11	122 ± 14	0.77
DBP (mm Hg)	73 ± 7	74 ± 6	0.45
Baseline 10-yr CVD risk (Framingham)	18.6 ± 9.1	21.1 ± 10.0	0.35
Baseline QRISK2	21.9 ± 9.5	20.5 ± 9.1	0.67
Creatinine (mmol/l)	79.2 ± 13.7	74.6 ± 13.9	0.85
Creatinine clearance (ml/min)	90.6 ± 22.0	97.9 ± 25.0	0.15
eGFR (ml/min/1.73 m^2^)	86.3 ± 15.0	87.5 ± 16.0	0.65
Total cholesterol (mmol/l)	4.5 ± 1.0	5.5 ± 0.8	0.008
HDL (mmol/l)	1.6 ± 0.4	1.6 ± 0.5	0.82
Uric acid (mmol/l)	0.41 ± 0.1	0.44 ± 0.1	0.08
BNP (pg/ml)	7.9 ± 3.8	21.0 ± 9.6	0.0001
hs-TnT (ng/l)	4.9 ± 2.1	6.9 ± 2.4	0.04
ACEi	12 (46%)	13 (54%)	0.41
ARB	8 (31%)	6 (25%)	0.51
Diuretics	10 (38%)	8 (33%)	0.88
Beta-blockers	5 (19%)	3 (12%)	0.49
Ca^2+^ channel blockers	10 (38%)	9 (37%)	0.86
Statins	16 (61%)	12 (50%)	0.38
Transmitral E/A	0.83 ± 0.20	0.84 ± 0.18	0.93
Transmitral E/e′	9.4 ± 2.1	9.7 ± 2.6	0.62
CMR baseline LVM	108 ± 21	103 ± 26	0.50
CMR baseline LVM index	56 ± 8	54 ± 9	0.54
CMR LVEF (%)	75.3 ± 5.0	75.8 ± 4.7	0.76
CMR LVEDV	122 ± 23	118 ± 33	0.13
CMR LVESV	31 ± 10	29 ± 11	0.30
Follow-up time (months)	36.2 ± 0.3	36.5 ± 1.2	0.42

Values are mean ± SD or n (%).

ACEi = angiotensin-converting enzyme inhibitor; ARB = angiotensin receptor blocker; BMI = body mass index; BNP = B-type natriuretic peptide; CMR = cardiac magnetic resonance; CVD = cardiovascular disease; DBP = diastolic blood pressure; E/A = ratio of the early to late ventricular filling velocities; E/e′ = ratio of the early diastolic transmitral flow velocity to the mitral annular velocity; eGFR = estimated glomerular filtration rate; HDL = high-density lipoprotein; hs-TnT = high-sensitivity troponin T; LVEDV = left ventricular end-diastolic volume; LVEF = left ventricular ejection fraction; LVESV = left ventricular end-systolic volume; LVM = left ventricular mass; SBP = systolic blood pressure.

∗ Student *t* test, Chi-square, Fisher exact test, or Mann-Whitney *U* test.

**Table 2:** Changes in CMR Data According to Baseline BNP Tertiles∗

CMR Variable	Tertile I (n = 16)	Tertile II (n = 17)	Tertile III (n = 17)	p Value†	p_TREND_‡
Change in LVM	–5.14 ± 2.64	–1.60 ± 4.67	5.49 ± 3.78	0.0001	0.0001
% Change in LVM	–5.04 ± 2.62	–1.16 ± 4.36	5.50 ± 3.79	0.0001	0.0001
Change in LVM Index	–2.92 ± 2.20	–0.98 ± 0.96	3.10 ± 2.87	0.0001	0.0001
% Change in LVM index	–5.24 ± 4.05	–1.80 ± 1.82	5.97 ± 4.77	0.0001	0.0001
Change in LVEF (%)	–0.18 ± 4.86	–1.15 ± 3.39	1.04 ± 2.83	0.28	0.38
Change in LVESV	1.31 ± 7.45	0.43 ± 5.40	–1.58 ± 4.01	0.53	0.37
Change in LVEDV	3.5 ± 11.2	–2.68 ± 10.18	–2.8 ± 10.90	0.33	0.35

Values are mean ± SD.

Abbreviations as in Table 1.

∗ Tertiles for n = 50: Tertile I 5.0 to 6.4 pg/ml, Tertile II 6.9 to 15.5 pg/ml, Tertile III 18.6 to 37.5 pg/ml.

† Kruskal-Wallis test.

‡ Jonckheere-Terpstra test.

No significant differences were noticed in demographics and prevalence of underlying primary risk factor(s) between the 2 groups except that the patients in whom an increase in LVM was observed were significantly more likely to be active smokers (41% vs. 12%, p = 0.02) or have higher cholesterol levels (5.5 ± 0.8 vs. 4.5 ± 1.0, p < 0.01). No significant differences were noticed in baseline BP as assessed by 24-h ambulatory BP monitoring, underlying renal function, or baseline pharmacotherapy, and baseline LVM was also similar in both groups at baseline (Table 1). The baseline diastolic parameters on 2-dimensional echocardiography, including the ratio of the early diastolic transmitral flow velocity (E) to the mitral annular velocity (e′), or transmitral E/e′, were not statistically different between those with or without a future rise in LVM.

### BNP and high sensitivity cardiac troponin T and change in LVM

**Figure 1:**
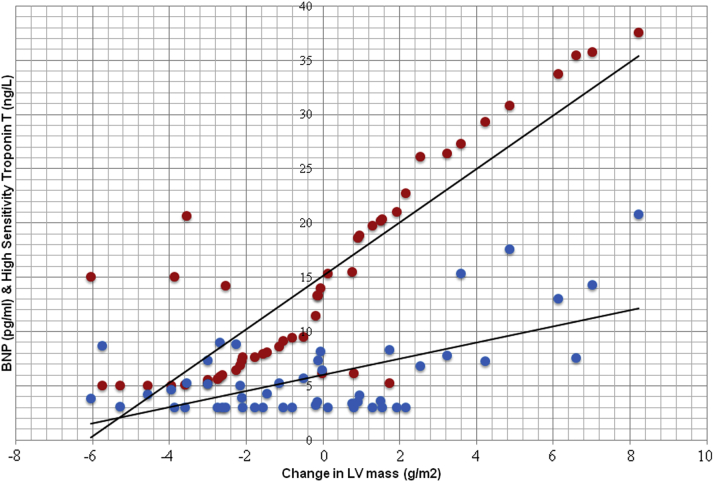
Distribution of Baseline BNP and hs-TnT Levels Across the Range of ΔLVM Red dots represent B-type natriuretic peptide (BNP) and blue dots represent high-sensitivity troponin T (hs-TnT). ΔLVM = change from baseline in left ventricular (LV) mass.

**Figure 2:**
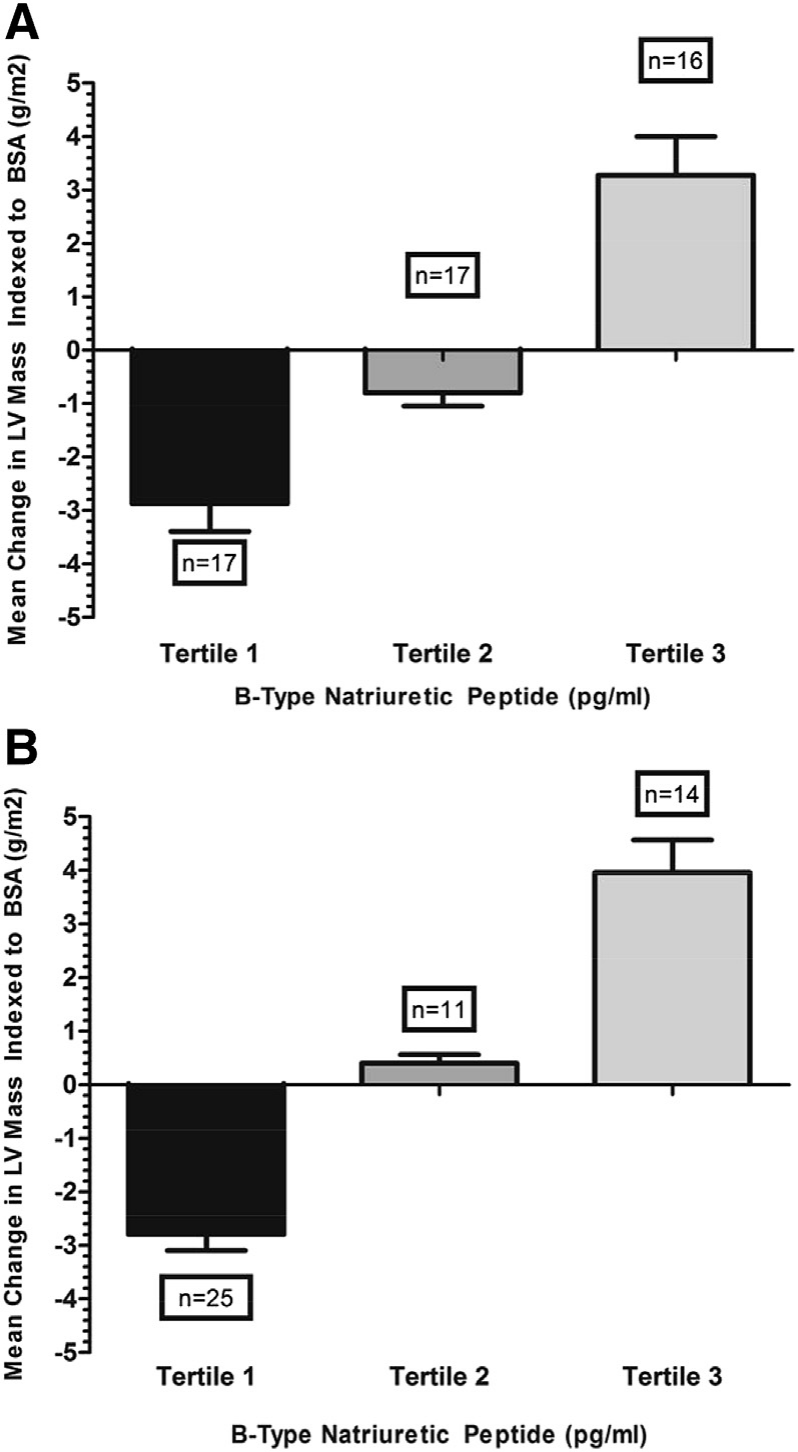
ΔLVM Across the BNP Tertiles The top diagram **(A)** uses the BNP tertile levels of this n = 50 cohort; the lower diagram **(B)** uses the BNP tertile levels of the original larger n = 300 cohort (1). Tertiles for n = 50: tertile I 5 to 6.40 pg/ml, tertile II 6.9 to 15.5 pg/ml, tertile III 18.6 to 37.5 pg/ml. BSA = body surface area; LVM = left ventricular (LV) mass.

Both BNP (mean BNP 21 vs. 7.9 pg/ml) and hs-TnT (mean hs-TnT 6.9 vs. 4.9 ng/l) levels at baseline were significantly higher in patients whose LVM increased with time (Table 1). There was no significant effect of age or sex on this observation. Among all the CMR parameters, only LVM and LVM index (LVMI) changed significantly according to baseline BNP values (Online Table 3). Correlation analysis showed a strong linear relationship (r = 0.71, p < 0.01) between ΔLVM and baseline BNP levels (Figure 1) There was a strong positive relationship between BNP tertiles and ΔLVM (Figure 2, Online Table 3). This was the case whether the BNP tertiles were calculated on the basis of the tertiles of the original study (n = 300) or the tertiles of this substudy (n = 50) (Figure 2). It is worth noting that the difference between tertile 1 and tertile 3 is large at nearly 12% of the mean baseline LVMI.

The independent predictive value of the interaction between ΔLVM and baseline BNP levels for explaining the evolution of LVM with time (dependent variable) was investigated by multiple linear regression analysis. We investigated 5 different models (Table 3). Model 1 was composed of previously reported clinical predictors of LVM such as age, sex, BP, body mass index, and history of smoking. Subsequent models explored the additional predictive value of adding total cholesterol and uric acid and then adding baseline hs-TnT or BNP on top of model 1. As shown in Table 3, both hs-TnT and BNP offered additional predictive value when added to the model by improving the c-statistics significantly. In a logistic regression analysis, BNP stood out as a strong predictor of a future rise in LVM. A receiver-operating characteristic analysis yielded a c-statistic of 0.88 for BNP with a sensitivity and specificity of 70% and 88%, respectively, at a BNP level of 17 pg/ml.

**Table 3:** Multivariate Prediction Models for Evolution of LVM

Model	c-statistics∗	p Value∗
Model 1: age, sex, SBP, BMI, and smoking	0.72	Model 1 vs. Model 2, p = 0.32
Model 2: age, sex, SBP, BMI, smoking, uric acid, and total cholesterol	0.76	Model 1 vs. Model 3, p = 0.11 Model 1 vs. Model 4, p = 0.0007
Model 3: age, sex, SBP, BMI, smoking, uric acid, cholesterol, and troponin	0.83	Model 1 vs. Model 5, p = 0.0009 Model 2 vs. Model 3, p = 0.27 Model 2 vs. Model 4, p = 0.001
Model 4: age, sex, SBP, BMI, smoking, uric acid, cholesterol, and BNP	0.98	Model 2 vs. Model 5, p = 0.002 Model 3 vs. Model 4, p = 0.01
Model 5: age, sex, SBP, BMI, smoking, uric acid, cholesterol, troponin, and BNP	0.98	Model 3 vs. Model 5, p = 0.01 Model 4 vs. Model 5, p = 0.01

Abbreviations as in Table 1.

∗ Pairwise comparison between the various models by DeLong method.

## Discussion

Our main finding is that, in well-controlled primary prevention patients, a high BNP in the absence of any cardiac abnormality is able to identify those individuals whose LVM will increase during the next 3 years, that is, an elevated BNP is able to predict *future* increases in LVM. This may partly explain why, in so many studies, BNP predicts prognosis independent of echocardiographic abnormalities.

The Framingham study has already shown that the tendency for LVM to increase with aging in a population is highly variable from one individual to the next 11, 12. Increases in LVM in treated hypertension are, however, far from innocent (13). Serial changes in LVM predict CV events, independent of baseline LVM and independent of baseline BP or the degree of BP reduction (14). It now appears from our data that BNP can identify those whose serial LVM will increase with time, and we know that such individuals are at increased risk and that they are currently inadequately identified by either baseline LVM or any BP parameter (14).

A major strength of our study is that the population studied was comprehensively phenotyped at baseline, that is, they were all assessed for LVM, LV systolic dysfunction, left atrial enlargement, LV diastolic dysfunction, and most importantly, for silent myocardial ischemia. All 5 of these abnormalities are known to increase BNP and to herald a poor prognosis 1, 15. However we know with certainty that in the population we studied here, none of these cardiac abnormalities were present at baseline, meaning that the BNP elevation at baseline was truly unexplained by any prevalent cardiac abnormality. This makes our study unique as most data on BNP being of prognostic significance over and above echocardiographic abnormalities did not do the comprehensive phenotyping that we did and in particular did not screen for myocardial ischemia, which is known to independently increase BNP 4, 5, 6, 7, 16. In previous studies, some of the prognostic significance of BNP over and above resting echocardiographic abnormalities could be attributable to silent myocardial ischemia, which was not tested for in those studies. However, we have here demonstrated an additional explanation for why BNP is prognostic beyond echocardiographic abnormalities at baseline, which is that baseline BNP can identify those whose LVM will increase with time.

A lot of recent data are suggesting that measuring BNP might one day establish a role for itself in better managing patients with treated hypertension (2). Paget et al. showed that there was a 3-fold increase in mortality in the top versus the bottom tertile of *N*-terminal pro-BNP in treated hypertensive patients, even after adjusting for traditional risk factors (2). Recent information from the ASCOT trial shows the same (17). These 2 papers suggest that BNP could become a measure within individuals at target BP of whether antihypertensive therapy will actually prevent CV events in them or not. In turn, this begs the question of which (treatable) cardiac abnormalities might be causing the residual risk seen in treated hypertensive patients with a high BNP. Nadir et al. answered that question by showing that treated hypertensive patients with a high BNP had a combination of LVH, LV diastolic dysfunction, LA enlargement, LV systolic dysfunction, and silent ischemia (in that order of frequency) (1). Importantly, many patients had multiple silent cardiac abnormalities. This study extends that information to now show that in those with no cardiac abnormality at baseline, the elevated residual risk identified by BNP is likely to be also related to *future* increases in LVM 18, 19.

There are a few likely explanations for our results. A time effect is likely in that a raised intracardiac pressure genuinely precedes the increase in LVM. BNP is a much more sensitive marker for this increased intracardiac pressure owing to its greater reproducibility of repeat measures and a greater measurement range, whereas imaging parameters change over a much smaller range. This means an early subtle elevation in intracardiac pressure can be picked up by BNP before LV abnormalities are either present or detectable on imaging. Although it is also plausible that an elevated BNP marker could merely be a marker of noncompliance, it is important to note that our study population had well-controlled primary risk factor at the baseline.

At this time, there are clearly no therapeutic implications for this work, but looking to the future, there could be. For example, aggressive risk factor control guided by BNP levels may prevent future development of LVH and by doing so it may even prevent a future CV event itself. The expression “Prevention is better than cure” is highly relevant here because a life-changing CV event related to LVH (such as a stroke or even sudden death) could occur while there is LVH before its regression is achieved, and full regression does not always occur anyway. The treatments that are known to regress *established* LVH in a normotensive patient (e.g., a lower target BP, aldosterone blockade) might one day be useful to prevent LVH from developing in susceptible patients already at target BP 20, 21. This notion of using BNP-guided aggressive risk factor control to prevent future CV events is supported by the results of the STOP-HF trial (22). In this trial a 42% relative risk reduction in the development of LV systolic dysfunction (with or without overt heart failure) was seen with intensification of therapy in patients with CV risk factors and a BNP >50 pg/ml. Moreover, it is also conceivable that the use of novel CMR techniques to characterize the underlying tissue changes seen in the evolution of LVM may help to identify new therapeutic targets in future.

### Study limitations

As ever, there are limitations to our study. The number of individuals is relatively low (n = 50); however, it uses CMR scanning, which is more sensitive than echocardiography. In fact, the mean difference between the top and bottom BNP tertiles at the end of 3 years was fairly large, nearly 12% of baseline LVMI. Moreover, this is a longitudinal study of the same individuals over time, which is more informative at understanding natural history than commonly performed cross-sectional studies. Second, the fact that we preselected patients across a relatively wide range of BNP values at baseline with no serial measurements with time may have flattered our results, although this was not an unreasonable approach to maximize the cost-effectiveness of our study because our primary aim or hypothesis was to see how individuals with high BNP levels differed over time from individuals with low BNP levels. This selection limitation is assuaged by 2 factors. First, the demography of our chosen cohort was virtually identical to the demography of the patients in our index study who had no target organ damage at baseline. Second, we used BNP tertiles from our index as well as the current study. These 2 added analyses strongly suggest that selection bias did not influence our results, although future research with larger numbers would be required to confirm these findings. Finally, BNP (and hs-TnT) levels seen in our study (not unexpectedly) are at the lower end of the range, and imprecision of the assay may be relevant at these levels.

## Conclusions

It now appears that optimally treated hypertensive patients with a top tertile BNP but a normal echocardiographic study are likely to experience an increase in LVM. This may in part explain why patients with high BNP levels and a normal echocardiographic study have a poor prognosis, including why they often experience atrial fibrillation and heart failure. The next stage would be tissue characterization with novel CMR techniques in the evolution of LVM and to see whether treatments that are known to regress established LVH can actually prevent LVH from developing in those identified to be at high risk by their having an otherwise unexplained high BNP.
