# Supplementary material for: An Increased B-Type Natriuretic Peptide in the Absence of a Cardiac Abnormality Identifies Those Whose Left Ventricular Mass Will Increase Over Time
Source: JACC Heart Fail. 2015 Jan;3(1):87–93. doi: 10.1016/j.jchf.2014.07.012 (PMC4286125; doi:10.1016/j.jchf.2014.07.012)

| Variables | BNP < 10 pg/ml  N = 25 | BNP > 10 pg/ml  N = 25 | Significance* |
| --- | --- | --- | --- |
| Age | 63.9 ± 6 | 61.5 ± 4.5 | 0.58 |
| Male/female | 17/8 (68%/32%) | 15/10 (60%/40%) | 0.74 |
| Hypertensive | 23 (89%) | 20 (80%) | 0.86 |
| Dyslipidemia | 18 (72%) | 13(52%) | 0.07 |
| Smoker | 5 (20%) | 8 (32%) | 0.05 |
|  |  |  |  |
| BMI | 26.5 ± 3.8 | 27.6 ± 3.1 | 0.29 |
| 24-hr ambulatory SBP | 122 ± 11 | 122 ± 15 | 0.89 |
| 24-hr ambulatory DBP | 73 ± 7 | 74 ± 6 | 0.96 |
|  |  |  |  |
| Duration of treatment | 3.7 ± 2.4 | 4.1 ± 4.7 | 0.65 |
| Baseline 10 year CVD risk (Framingham) | 19 ± 10 | 21 ± 9 | 0.43 |
| Baseline QRISK2 | 22 ± 10 | 21 ± 9 | 0.65 |
|  |  |  |  |
| Creatinine | 76 ± 13 | 76 ± 175 | 0.91 |
| eGFR | 87 ± 15 | 88 ± 17 | 0.32 |
| Total cholesterol | 4.7 ± 1.1 | 5.3 ± 0.9 | 0.40 |
| HDL | 1.6 ± 0.4 | 1.6 ± 0.5 | 0.85 |
| Uric acid | 0.41 ± 0.1 | 0.45 ± 0.1 | 0.07 |
| BNP^†^ | 6 (4.5) | 21 (13.8) | 0.0001 |
| hs-TnT^†^ | 4.5(5.9) | 7.5(4.7) | 0.04 |
|  |  |  |  |
| ACEi | 14 (56%) | 11 (44%%) | 0.41 |
| ARB | -6 (24%) | 8 (32%) | 0.51 |
| Diuretics | 9 (36%) | 9 (36%) | 0.65 |
| β-blockers | 4 (16%) | 4 (16%) | 0.94 |
| Ca^2+^ channel blockers | 9 (36%) | 10 (40%) | 0.44 |
| Statins | 16 (64%) | 12 (48%) | 0.26 |
|  |  |  |  |
| Transmitral E/A | 0.80 ± 0.2 | 0.85 ± 0.18 | 0.94 |
| Transmitral E/e′ | 9.1 ± 2.1 | 9.6 ± 2.6 | 0.92 |
|  |  |  |  |
| CMR baseline LVM | 107 ± 22 | 105 ± 26 | 0.71 |
| CMR baseline LVMI | 56 ± 8 | 54 ± 10 | 0.32 |
|  |  |  |  |
| Follow-up (months) | 36 ± 0.6 | 37 ± 1.3 | 0.11 |

ONLINE APPENDIX

Online Table 1. Baseline Characteristics of the Study Population by Median BNP (10 pg/ml)

*Student *t* test, Chi square, Fisher exact test or Mann-Whitney *U* test.

†Median with interquartile range.

BMI = body mass index; SBP = systolic blood pressure; DBP = diastolic blood pressure; CVD = cardiovascular disease; eGFR = estimated glomerular filtration rate; HDL = high-density lipoprotein; BNP = B-type natriuretic peptide; hs-TnT = high-sensitivity troponin T; ACEi = angiotensin-converting enzyme inhibitor; ARB = angiotensin receptor blocker; CMR = cardiac magnetic resonance; LVM = left ventricular mass; LVMI = left ventricular mass index.

Online Table 2. Demography of CMR Scanned 50 Patients Versus the 148 Patients (Out of 198 Excluding CMR Scanned 50 Patients) With No Cardiac Target Organ Damage

|  | N = 148 | N = 50 | Significance |
| --- | --- | --- | --- |
| Age | 63 ± 6 | 64 ± 6 | 0.56 |
| Male/female | 87/60 (59%/41%) | 32/18 (64%/36%) | 0.77 |
| Hypertensive | 132 (89%) | 43 (86%) | 0.80 |
| Dyslipidemia | 88 (59%) | 31(62%) | 0.91 |
| Smoker | 22 (18%) | 13 (26%) | 0.20 |
|  |  |  |  |
| BMI | 28 ± 3.8 | 27 ± 3.5 | 0.03 |
| 24 hr ambulatory SBP | 116 ± 18 | 119 ± 19 | 0.25 |
| 24 hr ambulatory DBP | 70 ± 15 | 72 ± 11 | 0.12 |
|  |  |  |  |
| Baseline 10 year CVD risk (Framingham) | 21 ± 11 | 19 ± 9 | 0.38 |
| Baseline QRISK2 | 23 ± 10 | 22 ± 9 | 0.26 |
|  |  |  |  |
| Creatinine | 75.2 ± 18 | 74.1 ± 17.5 | 0.33 |
| eGFR | 88 ± 17 | 88 ± 16 | 0.95 |
| Total cholesterol | 4.9 ± 1.1 | 4.7 ± 1.4 | 0.59 |
| HDL | 1.4 ± 0.5 | 1.5 ± 0.5 | 0.34 |
| Uric acid | 0.44 ± 0.1 | 0.42 ± 0.1 | 0.26 |
| BNP^†^ | 10.6 (11) | 14.2 ± 9.8 | 0.75 |
| hs-TnT^†^ | 4.5(2.7) | 5.5 ± 3.6 | 0.15 |
|  |  |  |  |
| ACEi | 60 (41%) | 25 (50%) | 0.49 |
| ARB | 34 (23%) | 14 (28%) | 0.53 |
| Diuretics | 59 (40%) | 18 (36%) | 0.56 |
| β-blockers | 22 (15%) | 8 (16%) | 0.95 |
| Ca^2+^ channel blockers | 56 (38%) | 19 (38%) | 0.57 |
| Statins | 88 (59%) | 28 (56%) | 0.39 |

*Student *t* test, Chi square, Fisher exact test or Mann-Whitney *U* test.

†Median with interquartile range.

BMI = body mass index; SBP = systolic blood pressure; DBP = diastolic blood pressure; CVD = cardiovascular disease; eGFR = estimated glomerular filtration rate; HDL = high-density lipoprotein; BNP = B-type natriuretic peptide; hs-TnT = high-sensitivity troponin T; ACEi = angiotensin-converting enzyme inhibitor; ARB = angiotensin receptor blocker; CMR = cardiac magnetic resonance.

Online Table 3. Changes in CMR Data Divided by Evolution of LVM

| CMR Variable | LVM Decreased (n = 26) | LVM Increased (n = 24) | Significance |
| --- | --- | --- | --- |
| Change in LVM | –4.9 ± 2.8 | 4.7 ± 3.5 | 0.0001 |
| % Change in LVM | –4.7 ± 2.5 | 4.8 ± 3.5 | 0.0001 |
| Change in LVM index | –2.2 ± 2.0 | 2.0 ± 3.1 | 0.0001 |
| % Change in LVM index | –3.9 ± 3.6 | 3.7 ± 5.4 | 0.0001 |
| Change in LVEF% | –0.75 ± 4.3 | 0.39 ± 3.4 | 0.24 |
| Change in LVESV | –1.4 ± 6.7 | –1.1 ± 4.5 | 0.18 |
| Change in LVEDV | 2.3 ± 11 | –3.2 ± 10.4 | 0.04 |

CMR = cardiac magnetic resonance; LVM = left ventricular mass; LVEF = left ventricular ejection fraction; LVESV = left ventricular end-systolic volume; LVEDV = left ventricular end-diastolic volume.

Online Figure 1 Study flow diagram (Supplement files only)


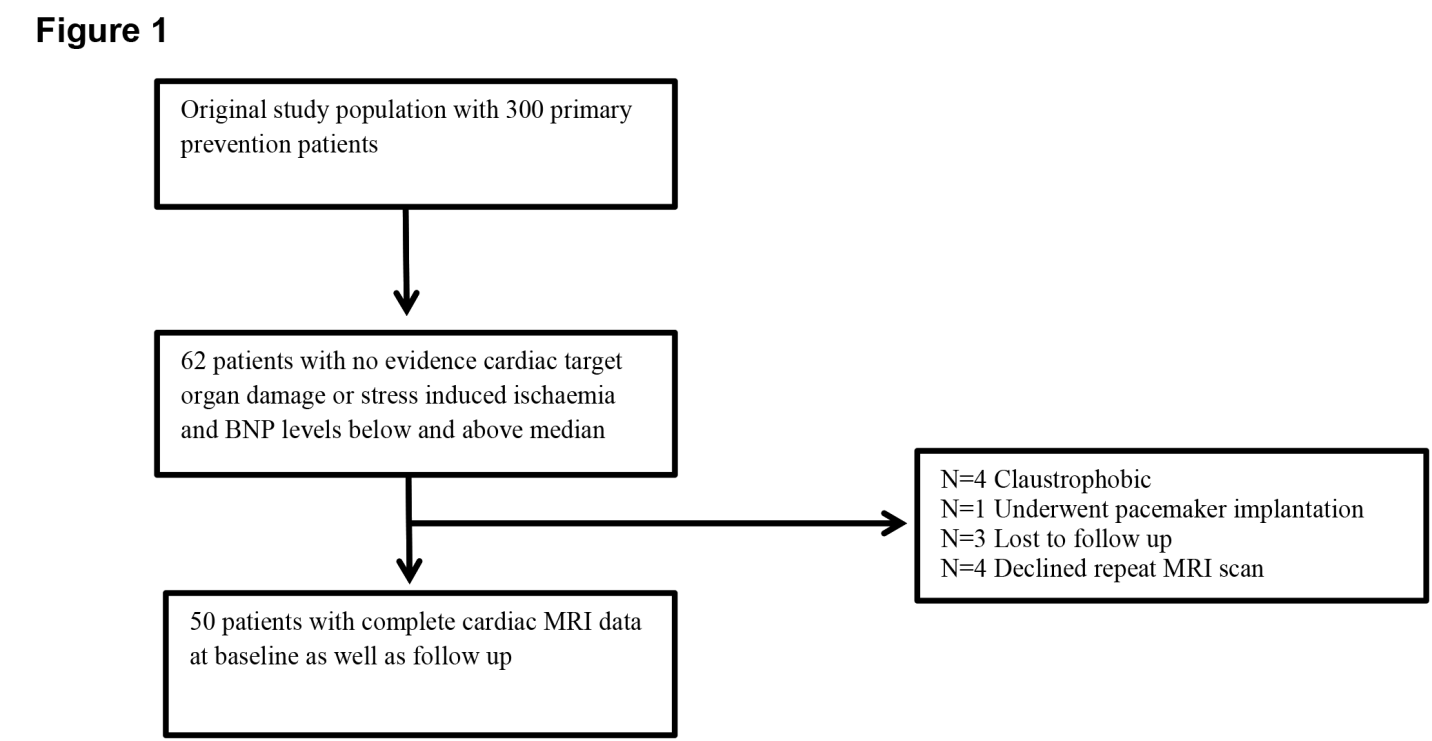

Supplement: Online Tables 1–3 and Online Figure 1 [file mmc1.docx]
